# Feasibility of an observational procedure to enhance early identification of autism spectrum disorder in paediatric settings: A mixed-methods study on an Ecuadorian sample

**DOI:** 10.1177/13623613231175587

**Published:** 2023-06-14

**Authors:** Paulina Buffle, Cristina Armijos, Alfredo Naranjo, Edouard Gentaz

**Affiliations:** 1 University of Geneva, Switzerland; 2 Pontificia Universidad Católica del Ecuador, Ecuador; 3 Universidad de los Hemisferios, Ecuador

**Keywords:** autism spectrum disorder, context-dependent tasks, early identification, low- and middle-income countries, paediatric settings

## Abstract

Identification procedures for autism spectrum disorders in paediatric settings include screening and routine clinical surveillance. Screening tools are perceived as challenging, and a preference for direct observation has been reported in Ecuadorian paediatric settings. Augmentative observational procedures could prompt the application of screening tools, increase professionals’ sense of self-efficacy and support referral decision-making. Following the recommendations that identification procedures should be tested in a group of children without autism from local populations, we tested a set of observational tasks on 125 children from 12 to 59 months of age in various socio-economic settings, in semi-experimental conditions and on a group of 33 non-autistic children in a paediatric-setting condition. Results indicated that the social referencing task did not produce the expected response in most children in semi-experimental conditions. Responses to the elicitation of a gesture were significantly influenced by socio-economic status and geographic area. Three other tested tasks (response to name, eye contact, response to joint attention) produced the expected responses in semi-experimental and clinical conditions, suggesting that they could be used in daily routines if fidelity to training is ensured. We discuss the implication of those findings in clinical practice and professional education in an Ecuadorian context.

**Lay abstract:**

In Ecuador, the low official estimate of the number of persons with autism spectrum disorder suggest that many children are not identified and are not receiving support. Screening tools are short parent-addressed questionnaires used to identify children that may be developing with autism. Their use is recommended, but their application can be perceived as challenging in paediatric routines. Some professionals prefer looking for autism-related behaviours in a child rather than using screening questionnaires. Although a short observation does not replace the use of validated screening questionnaires, tasks to guide the observation of autistic early signs can help professionals decide to screen or refer the family for assessment and early intervention. In this study, we tested observational tasks that could be adapted to the Ecuadorian paediatric contexts.

## Background

Studies on the prevalence of autism spectrum disorders (ASDs) (*Diagnostic and Statistical Manual of Mental Disorders* (5th ed.; DSM-5); American Psychiatric Association [APA], 2013) in various regions of the world suggest that at least one in one-hundred children develops with this condition (Elsabbagh et al., 2012; Shaw et al., 2020; Zeidan et al., 2022). Identifying individuals with ASD is required to inform health and education policies, respond to families’ concerns and provide information on autistic individuals’ needs and abilities. Although support at any age may be necessary, ASD-specialized interventions for very young children are important because they can represent the best opportunity to maximise developmental outcomes (Fuller & Kaiser, 2020; Sandbank et al., 2020; Vivanti et al., 2016; Zwaigenbaum et al., 2015). Behavioural characteristics of ASD usually emerge between 12 and 24 months of age (Barbaro & Dissanayake, 2017; Zwaigenbaum et al., 2019). However, many factors at the child, family and community levels have been associated with delays in detection, including greater symptom severity, lower socio-economic status, ethnic minority status, low caregiver awareness of the early signs of autism and visiting more significant numbers of health providers before diagnosis (Mandell et al., 2005).

In many countries, as is the case in Ecuador, professionals working in paediatric settings (paediatricians, family and community doctors) are in constant contact with young children and their families (Ministry of Public Health, 2018) and can play a critical role in case identification. Early identification practices in paediatric settings include developmental surveillance and detection. Developmental surveillance is defined as recognizing a child developing with a delay during well-child visits. In the case of socio-communicative development, surveillance strategies can include calling a child by his name and noting the responses or using specific presses to obtain joint attention, such as touching the child’s shoulder, pointing and exclaiming ‘Look!’ (Johnson & Myers, 2007; Zwaigenbaum et al., 2015). Detection implies the use of standardised tools to refine the observation of an increased likelihood of developmental delay. In the case of ASD, it includes applying specific tools, mostly parent-addressed questionnaires, at 18 and 24 months of age, after clinical observation and whether or not parents spontaneously raise concerns (Johnson & Myers, 2007; Zwaigenbaum et al., 2015). Several barriers to identification practices have been reported in paediatric settings. Some barriers include professional’s lack of familiarity with screening tools and limited time available for their application (Dosreis et al., 2006), difficulties to identify tools adapted to a child’s age and situation (Fenikilé et al., 2015), insufficient training (Morelli et al., 2014) and low self-perceived sense of efficiency to clinically identify signs of autism (Pinto-Martin et al., 2005). In addition, in some contexts, professionals may prefer a general clinical check-up without a screening instrument, or use formal screenings only if parents express concerns, a situation that can be problematic knowing that not all parents identify the early characteristics of ASD (Buffle & Gentaz, 2019; Dosreis et al., 2006; Sand et al., 2005).

Early ASD case identification should lead to a complete assessment and intervention when these services are available. In a context where those services are not readily available, reliable and contextually adapted identification procedures in paediatric or community services can lead to the characterisation of a child’s needs, identifying potential co-occurring conditions and guiding the family. In low- and middle-income countries (LMICs), routine identification procedures can be an important first step towards addressing families’ requirements for ASD services (Marlow et al., 2019). Many efforts have been deployed to enhance the application of validated ASD-screening tools, such as parent-addressed questionnaires (Marlow et al., 2019; Stewart & Lee, 2017). However, in some cases, these screening tools may suffer from limitations, such as the use of non-validated translations (Scarpa et al., 2013), reduced reliability due to lack of content adaptation to some non-Western cultures (Perera et al., 2009), poor parental literacy levels, lack of knowledge about milestones and social stigma influencing parental responses (Marlow et al., 2019). Improving screening practices and documenting ASD case identification can also provide health authorities with crucial data to understand the needs of the autistic community to plan and monitor further services.

During the past 30 years, studies on the developmental trajectories of ASD-characteristic behaviours have identified ways to elicit specific socio-communicative behaviours aiming to observe a child’s response, rather than relying on spontaneous occurrences (Baird et al., 2000; Barbaro & Dissanayake, 2009; Baron-Cohen et al., 1992; Lord et al., 2000; Mundy et al., 2003; Robins et al., 2001; Rutter et al., 2003). Transferring this expertise into clinical and community paediatric contexts could prove critical for developing clinical knowledge and a sense of self-efficacity in ASD identification.

Practice Improvement Approaches, such as augmentative procedures, aim to introduce quality improvement strategies into health providers’ daily routines to enhance screening and referral (Daniels et al., 2014), intending to expand professionals’ knowledge and sense of self-efficiency. Previous results in non-Western settings have denoted the potential of augmentative procedures based on the elicitation of ASD-characteristic behaviours, tested in local populations and aiming to inform clinical decisions and promote screening practices. Researchers in Turkey, for example, have tested an augmentative ASD-screening procedure on a group of children aged from 16 to 60 months with autism (*n* = 86), with developmental delay without ASDs (*n* = 76) and children without developmental disorders (*n* = 97). Using a three-item direct observation procedure (*Joint Attention, Eye Contact* and *Responsiveness to Name*) and the parent’s responses to items from the Social Communication Questionnaires (SCQs) (Allen et al., 2007), researchers found a sensitivity and specificity of 0.82 and 0.90 for *Joint Attention*, 0.89 and 0.91 for *Eye Contact* and 0.67 and 0.87 for *Responsiveness to Name*, to detect children with ASD. Their results also indicated sensitivity ratings of 0.073 and specificity of 0.70 for the diagnosis of autism with the SCQ. The authors suggested that using observational items completed by trained paediatric-oriented professionals could be a highly effective tool in improving screening performance in primary care (Oner et al., 2013). In a subsequent study, using the same observational procedure, researchers found this three-item procedure to be more sensitive and have a higher PPV than M-CHAT in 511 children (ages 16–36 months) from the general population. Based on these results, the authors suggest the combination of parent reports and the observational procedure for toddlers attending well-child clinic visits (Topçu et al., 2018). Another study, performed in China on a group of 212 children with mental ages between 18 and 24 months (Wong et al., 2004), used a combination of four observational items from the Checklist for Autism in Toddlers (CHAT) (Baron-Cohen et al., 1992) (*Eye Contact, Gaze Monitoring, Pretend Play* and *Protodeclarative Pointing*) and the 23 questions directed to parents of the M-CHAT (Robins et al., 2001). Authors found that an optimal cut-off point for differentiating between autism and non-autism was failing any 6 of 23 questions or failing any 2 of what they found to be the 7 most discriminative questions from the M-CHAT. They also found that failing any two of the four observational items produced a sensitivity of 0.736, specificity of 0.912 and PPV of 0.853. Based on these results, the authors recommend using the parental questionnaire followed by the observational procedure performed by trained evaluators, to identify children needing further assessment.

### Context

In Ecuador, the prevalence of ASD in a child population aged 5 years or less was estimated in 2017 at 0.28% (0.18%–0.41%), and 1266 people diagnosed with ASD were registered in official records (Ministry of Public Health, 2017). As preliminary evidence, a study aiming to estimate the attendance of children with an autism diagnosis in schools in Quito found a proportion of 0.11% among 453 pupils, ages 5–15, in 161 in regular schools (Dekkers et al., 2015). The reasons why Ecuador’s estimates are considerably lower compared to recent studies have not been clarified.

The health authorities recommend identifying children and adolescents at risk for a developmental disorder (Ministry of Public Health, 2018). Specific recommendations for ASD, published in a clinical guide in 2017, include the Denver Developmental Screening Test II in the first level of attention for children younger than 5 years. It also includes the use of a test or questionnaire for autism in young children for use between 16 and 30 months of age, containing one section (9 questions) for parents and one section (5 questions) for the clinician (Ministry of Public Health, 2017). This guide also refers to the M-CHAT, a questionnaire containing 23 parent-addressed items in its original version (Robins et al., 2001).

Although various screening instruments have been translated into Spanish (Alonso Esteban et al., 2020), in Ecuador, a first study of the perceptions of identification practices on a group of paediatric professionals (*n* = 39) has reported several barriers to screening practices and indicated that ‘direct observation of the child’s behaviour’ was perceived as the most adapted practice for their daily routine (42.86% of participants) (Buffle & Gentaz, 2019). Other identification procedures perceived as adapted were: ‘general developmental assessment tools’ (23.81%), ‘validated questionnaire in case of suspicion of a risk’ (14.29%) and ‘ask parents about their concern’ (11.90%). Only a small proportion of responses (7.14%) indicated that the use of ASD parent-addressed questionnaires was adapted to their usual practice. The participants also perceived ASD-specific questionnaires as ‘not useful if a child is showing a normal development’, ‘lack of reliability on parental responses’ and ‘non-comfortable’ or ‘difficult to find’ as the main drawbacks. A second study (Buffle et al., 2022), conducted on 183 paediatric professionals, indicated a low number of cases of autism identified during participant’s professional life, with 62% of participants reporting not having identified ASD cases during their professional life. This study also identified several barriers consistent with those identified in other countries, including limited knowledge of screening formal application, lack of time, lack of resources to refer and information to provide families, and fear of unnecessarily alarming families (Buffle et al., 2022).

### Aims

The study aimed to test the feasibility and adaptability of an augmentative procedure based on the observation of ASD early signs. Considering that identification procedures should be tested in a group of children without developmental difficulties from local populations (Ertem & WHO, 2012), and based on the hypothesis that the evolutionary nature of elicited behaviours would leave no room for early environmental influences, we expected that children without ASD would provide an optimal response to the elicitation of a series of socio-communicative presses (Study 1). We also expected that the most adapted tasks in terms of time, material and comfort could be used in a clinical environment and that the professionals who apply these tasks could achieve fidelity to a brief training (Study 2).

## Methods

### Study 1

Informed by the literature, we established a list of early symptoms that can inform a clinician of an increased likelihood of ASD (Barbaro & Dissanayake, 2009; Barbaro et al., 2011; Boyd et al., 2010; Robins et al., 2009). Considering that negative symptoms in the social and communicative domains, such as decreased pointing, reduced eye contact and lack of symbolic play, are predominant (Filipek et al., 1999; Ventola et al., 2007), we exclude positive symptoms, such as repetitive behaviours, restricted interests and unusual sensory and motor behaviours, which can appear mainly after 2 years and could also hardly be elicited during a consultation (Barbaro & Dissanayake, 2009).

As the main objective of this study was to increase the chances of selecting meaningful and comfortable items for the final users, we required the opinion of 18 paediatric professionals (resident paediatricians, school doctors, family doctors) on the adaptability of items to their daily routine. We considered two main criteria: the applicability of the items in a short period and the use of readily available materials in clinical practices. A list of 12 items was proposed: elicitation to response to name; elicitation to joint attention; elicitation of a pointing gesture; elicitation of eye contact; elicitation of social referencing; elicitation of language understanding; elicitation of pretending play; elicitation of response to joint attention; elicitation of imitation; observation of protodeclarative pointing; observation of proto-imperative pointing; observation of the ability to show objects. Five items were endorsed as adapted to a daily routine by more than 50% of the participants (Table 2).

#### Procedure

The study was conducted in accordance with the latest Declaration of Helsinki and its experimental protocol had been approved by the Ethics Committee of the university where the first author is affiliated. To test the hypothesis that the five selected items would elicit expected responses in a group of preschool-age non-autistic children, we recruited a group of participants through public announcements addressed to preschools in the town of Quito and its surroundings. Preschools staff interested in participation contacted the investigator to receive information. While the final participation was decided to have a geographical distribution with urban and rural representation, as defined by the local administrative system, the school’s participation was decided on a first-come-first-served basis. Families interested in participating were informed of the study’s objectives, particularly our interest in studying socio-communicative behaviours in healthy children. They were also informed about the need to videotape their child’s responses. Parents signed an informed consent. Testing sessions were organised according to the availability of the parents and the institution. Parents responded to a demographic questionnaire. The socio-economic level was inferred from the cost parents pay for the preschool.

Participation of children without ASD was controlled with a questionnaire on developmental milestones and the Spanish versions of the M-CHAT (under 31 months) and the SCQ (children over 32 months). Both instruments were self-administered by caregivers with the researcher’s assistance. The level of communication was evaluated with the Language scale of the Bayley Development Scales and the Vineland Adaptive Behavior Scales (VABS; Sparrow & Cicchetti, 1989). At the end of the experiment, the principal investigator met with the parents of children who had scores above the cut-off points to provide information on the development of the children (all parents had given their prior written consent to be informed if difficulties were detected development during research).

Data were collected in a separate calm room in urban preschool centres and a community house in rural areas. Children were accompanied by a caregiver who sat behind or stood next to the child. The presentation time of each stimulus was of less than 1 min (In addition to the present research, an experimental study related to visual attention was conducted with the same participants using an independent task outside of this project.) All the children who participated received a wooden puzzle to thank them for their participation. The demographic characteristics of the 125 children who met the participation criteria were analysed (Table 1).

**Table 1.** Sample characteristics of 125 children in the semi-experimental setting in study 1.

Gender	
Male	48
Female	77
Age (in months)	
Age range	12.2–59.2
Age mean	33
Socio-economic level	
High and middle-high	64
Low and middle-low	61
Residency area	
Urban	77
Rural	48

### Data coding and results

The behavioural responses of 125 participants were analysed by double-coding video-recorded scenes by the experimenter and a local student on languages-arts, blind to the objectives and fluent in Spanish. The two codings were consistent (98.4%). When discrepancies occurred (two cases in total), a third coding, also blinded to the objectives (a school-based medical doctor), was required. Conflictual situations were solved by the majority. The very small number of discrepancies indicates that direct coding of behaviours by the person who is eliciting the task (in this case, the experimenter) is reliable. The reliability of simultaneous coding and administration of the tasks is essential, as a clinician would need to elicit the behaviours and code them at the same time. The social referencing item (introducing an ambiguous situation unexpectedly to observe a child’s orientation and adapting behaviour in function of adult’s emotional expression) implied using a video camera in a limited space and in a non-invasive way, which proved challenging. For this reason, responses were coded in real-time by the experimenter and a student nurse trained in the procedure. Rates of responses were calculated for the five items, and analyses of rate differences were performed according to the residency area and the socio-economic status.

As indicated in Table 2, most participants provided an optimal response (in one or two presses) to the four tasks. However, the social referencing task did not produce the expected response in most children. The high non-optimal response rate in a behaviour known to be impaired in children with ASD indicates that this task may not be an appropriate item to be included in an observational procedure in Ecuador. For this reason, the social referencing item was excluded from the second part of the procedure. We examined if the rates of children who provided optimal responses to the four remaining items depended on the socio-economic status. A chi-square test for independence indicated that the difference of rates is significative only for the item related to the elicitation of a pointing gesture, with a higher rate in high middle-income settings (81% vs 57% in low middle-income setting; χ^2^ = 8.41, *p* < 0.05).All other differences were not significant (*p* > 0.25).

**Table 2.** Rate of optimal responses to 5 behavioural tasks by 125 children in study 1.

Behavioural responses to each task	Rate (%)
Elicitation of eye contact	97
Elicitation of response to name	93
Elicitation of joint attention	96
Elicitation of a pointing gesture	70
Elicitation of social referencing (orientation)	18
Elicitation of social referencing (adapting behaviour)	4

We also examined dependence on the geographical area. A difference in the rate of urban and rural settings was calculated for the four behaviours with a non-parametric test (χ^2^ = 4.64, *p* = 0.031, *p* < 0.05). A chi-square test for independence indicated a significative link of the elicitation of a pointing gesture, with more gestures produced upon request in children from urban (77%) than from rural residency (58%). All other differences were not significant (*p* > 0.10).

**Figure 1.**
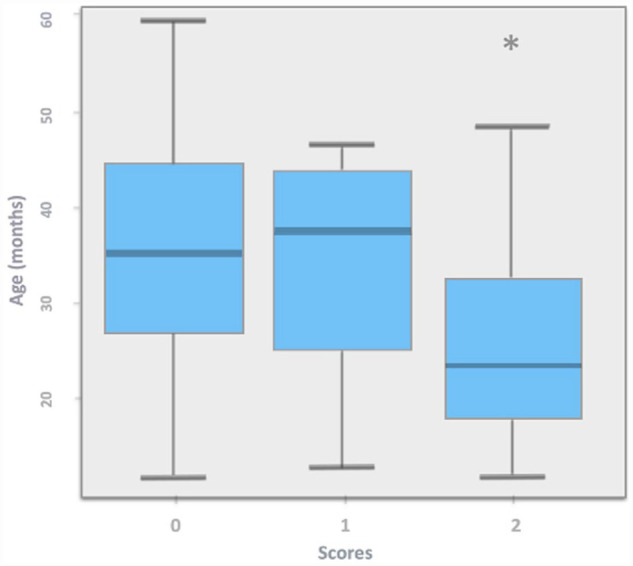
Scores distribution by age (in months) for the task `elicitation of a pointing gesture in study 1. The asterisk indicates the presence of an outlier.

A descriptive analysis was conducted to control a significant association between the scores (0, 1, 2) and age distribution (in months). It indicates the presence of differences between the distribution of age in each type of score only for the task related to the elicitation of a pointing gesture. For this task, a box plot indicates similarities between ages for scores 0 and 1, while score 2 corresponds to a lower age level (Figure 1). A scatter plot, indicating a large concentration of data for level 0, compared to scores 1 and 2, corroborates that the means of age for the group scoring 0 and 1 are similar but lower for the group scoring 2. A Shapiro–Wilk test (with an alpha level of 0.05) indicates that the variable age does not follow a normal distribution (*W* = 0.97125, *p* = 0.0090), and a Levene’s test for homogeneity of variance does not reject homoscedasticity (*t*(122), *d* = 2, *p* = 0.7886). This could indicate a certain level of homogeneity between groups. However, the difference in data concentration between the three groups and the presence of an outlier in group 2 does not assure homogeneity. A non-parametric test of Kruskal–Wallis was used to compare the means of age for three groups: 35 children (males = 17) from 12 to 36 months (M = 18.46 ± 0.61), 41 children (males 14) from 37 to 49 months (M = 30.91 ± 0.56) and 49 children (17 males) from 50 to 59 months (M = 46.39 ± 0.94) for each score. Results indicate that the groups are not homogeneous. To detect a significant difference, a post hoc analysis of multiple comparisons, based on confidence intervals with a Bonferroni test at 95%, indicates significative differences between groups for scores 0 and 2 (difference = 26.59, *p* = 0.0011). In this sample, the mean age of children obtaining a score of 0 (M = 35.97; SD = 11.93) was higher than the mean age of children obtaining a score of 2 (M = 27.09; SD = 11.42) and the mean age of children receiving a score of 1 (M = 33.87; SD = 12.78) does not significantly differ from those scoring 0 or 2. These results need to be interpreted with caution as the sample may not be representative.

However, a precise age cut-off for optimal response in this task could not be identified. A diagram of dispersion with age (in months) and optimum response (score 0) does not indicate the presence of clusters. To verify this, a goodness-of-fit test was used under the following analysis: if the age variable presents a grouping or cut-off point, its frequency distribution would be different in certain values of the scale. Also, if there is no cut-off point, the data distribution would be uniform. Some irregularity in the distribution is observed when performing the respective frequency histogram. However, in most cases, the frequencies are relatively close to what would be expected under a uniform distribution. A comparison of frequencies observed versus expected values of a uniform distribution, with a Kolmogorov–Smirnov test (*p* = 0.48), does not reject the possibility that the age distribution is uniform. Therefore, cut-off points could not be established between the data. However, a tendency of a higher proportion of responses could be seen around 36.2 months, with 80.8% of optimal responses compared to 61.6% for younger children. This limit was used as a reference to establish age categories.

## Study 2

This study aimed to test the hypothesis that the four tasks successfully implemented in the semi-experimental phase would produce the same rate of neurotypical responses on a group of children if used in a paediatric clinical setting. We expected a high-fidelity rate after a short training delivered to professionals. We also hypothesised that attitude towards the procedure among professionals should be positive in terms of time, material and comfort.

### Procedure

Paediatric professionals were enrolled through the paediatric school from a local university and public announcements to medical staff working within the educational settings (schools) and health settings (hospitals and private practices) to maximise diversity. A group of 12 professionals, ages 29–57 years, two general medical doctors working in a school setting, three interns in paediatrics, three paediatricians, two nurses working in preschool settings, one nurse working in paediatric settings and one paediatric physiotherapist were recruited on a first-come, first-served basis. Professionals were informed of the study’s objectives and received one-and-a-half-hour training before implementing the four-item procedure. The training was delivered in groups of two-to-three professionals. It contained a theoretical part (60 min) and a practical section where professionals were first shown videos (not related to any study) with the experimenter eliciting the tasks (15 min). Next, professionals had the opportunity to train the skills and receive feedback from the author to achieve fidelity (15 min). Training included information on how to code the observed behaviours.

Children we recruited through public announcements following the same procedure as in study 1. Parents and preschool staff received information about participation. Thirty-three families met the inclusion criteria and were recruited on a first-come, first-served basis (Table 3). Professionals and families were informed of the study’s objective and the need to video record the responses. All professionals and parents signed an informed consent. All participants, adults and children were informed that they could end their participation at any time. Sessions were organised in two paediatric services and one preschool’s nurse office, according to professionals’ and parents’ availability. Children were accompanied by a parent or a caregiver who remained next to the child, simulating a paediatric consultation. The time of presentation for each stimulus was less than 1 min. The task’s characteristics and protocol were the same as in Study 1, except for the elicitation of joint attention. For this item, as a very high proportion of children had provided an optimal response of orientation towards the first two clues (98%), we decided to use two instead of four cues to limit implementation time and raise the level of comfort of professionals. Families filled out a demographic questionnaire and responded to questions about their child’s development (M-CHAT or SCQ depending on the age and VABS). After the behavioural procedure, a questionnaire was submitted to paediatric professionals. Two questions addressed (1) their perceptions and attitudes towards the tasks in terms of time required, adaptability and comfort and (2) the likelihood of integrating this observation as a part of surveillance procedures.

**Table 3.** Sample characteristics of 33 children in a clinical setting in study 2.

Gender	
Male	13
Female	20
Age (in months)	
Age range	12.7–58.8
Age mean	37.8
Socio-economic level	
High and middle-high	19
Low and middle-low	14
Residency area	
Urban	20
Rural	13

## Results

### Influence of the setting on behavioural responses

We analysed the responses to 4 elicitations produced by 12 professionals in a group of 33 non-autistic children (13 males, 20 females) aged 12.70–51.80 months (mean = 37.79) from various socio-economic and residency backgrounds. All trials were video-recorded and simultaneously coded by the researcher and the professional participants. The video records served to compare to coding. No discrepancies occurred. The responses to the elicitation of four behaviours were compared to results obtained in the semi-experimental situation (Table 4).

**Table 4.** Difference of rates of optimal responses percent in function of two testing settings in study 2.

Tasks	Semi-experimental setting	Clinical setting	*p*-value
Elicitation of eye contact (blowing bubbles)	97%	62%	**0.0000**
Elicitation of response to name	93%	100%	0.0537
Elicitation of joint attention (pointing to two posters)	96%	100%	0.4074
Elicitation of a pointing gesture (asking a question)	70%	79%	0.3020

Bold value refers to p < 0.001.

### Fidelity

According to the training provided, this measure consisted of optimal implementation rates of predefined steps for each of the four tasks. Fidelity was defined as follows: 2 points indicating that the professionals were fully compliant with the action taught during training, 1 point if they were partially complying and 0 points if the action was not performed. Each of the four tasks required two or more steps as follows: elicitation of eye contact: (1) obtaining the child’s attention, (2) blowing the bubbles and (3) having eyes and face available for eye contact. Elicitation to response to name: (1) wait until the child is not orienting towards you and (2) call the child’s name. Elicitation to joint attention: (1) obtain the child’s attention and (2) orient your gaze and point towards the poster on your right and say ‘look’, (3) obtain the child’s attention a second time and (4) orient your gaze and point towards the poster on your left and say ‘look’. Elicitation of a pointing gesture: (1) obtaining the child’s attention, (2) asking the child to situate a specific object, for example, ‘Have you seen teddy, where is teddy with the red cap?’ and (3) specifically, asking the child to point to an object. Together, these 12 steps became the denominator for identifying the percentage of fidelity for implementation. Fidelity to the original procedure was based on the direct coding of all the test situations performed simultaneously by the experimenter and a local trained Spanish-speaker assistant (a student nurse). Three conflict situations were resolved based on videos. The percentage of optimal elicitation procedures (professionals scoring 2 points) was calculated.

The 4-item procedure was applied on 33 participants; in 28 cases, professionals reached at least 75% of fidelity, and 5 procedures had a fidelity rate between 50% and 70%. The lowest rate of optimal elicitations, among the 12 defined phases, corresponded to the following steps: obtaining children’s attention before eliciting eye contact with bubbles (48%) and obtaining children’s attention before joint attention towards poster number one (64%) and towards poster number 2 (58%).

### Attitudes and perceptions of the four tasks among professionals

All participants (1) perceived the four tasks as adapted to their practice in terms of time and comfort and (2) rated the likelihood of integrating them as part of their daily routine as high. Unfortunately, the participants were not blinded to the study’s objective; henceforth, a bias in their responses could be present. After analysing the opinions of professionals, it was found that they consider this procedure very useful to learn about the behaviours that are indicative of ASD in young children, as indicated by the following citations: ‘I would never have payed attention to those detailes if I hadn’t been made aware of them [Si no me dicen, nunca me habría fijado en esos detalles], ‘It is true that you need to know how to observe’, [Verdad que hay que saber que observar], ‘I did not know it was possible to observe those things with children’ [No sabía que se podia observar esas cosas en los niños].

## Discussion

This study aimed to test the feasibility and adaptability of five socio-communicative items corresponding to behaviours that have an atypical development in children with ASD. We suggest that among the items we tested, four are adapted to be used as an observational procedure. This procedure can play an important role in primary care routines. Combined with a short, validated and culturally adapted questionnaire, it can contribute to eliminating barriers to screening and identification that have previously been reported by Ecuadorian paediatricians (Buffle & Gentaz, 2019; Buffle et al., 2022).

Our results suggest that items’ adaptability could depend on various factors, such as the characteristics of the task or the material used. For instance, the meagre rate of optimal responses to the social referencing, task described as reduced or absent in children with ASD (Cornew et al., 2012; DeQuinzio et al., 2016), suggest that this item is not adapted to a clinical context in Ecuador. The efficacity of this item could depend on the type of object used, the room’s configuration or the assistance of a compere charged to introduce the object, a variety of factors that are difficult to control in paediatric practices. Further investigations, however, are necessary to clarify if social referencing develops differently in Ecuadorian children than in other contexts or whether the characteristics of the task need to be improved.

The results indicate that items’ adaptability could also depend on the socio-economic status or the residency area where a child develops. The elicitation of a pointing gesture, for example, produced a significantly higher proportion of optimal responses in children from a high and medium-to-high socio-economic level and in children living in urban areas. However, in this task, the effect of a non-familiar adult’s presence cannot be ruled out. Concerning age, the differences in the rate of responses among age groups were not significant for any of the items used. However, in the item elicitation of a pointing gesture, the mean age of children obtaining 0 (optimal response) tended to be higher than the mean age of children obtaining scores of 1 or 2. However, a precise cut-off for optimal response in terms of age could not be defined. Further investigations on the effect of the adult’s presence and children’s age are needed for this item. In the meantime, prudence in interpreting a lack of response in young children is recommended.

As expected, the elicitation of eye contact, response to name and joint attention produced a very high rate of optimal responses in the semi-experimental and clinical conditions. However, the elicitation of eye contact in the clinical situation produced significantly less optimal responses than in the semi-experimental situation. A low fidelity to training rate could partially explain this difference, particularly in attracting the child’s attention before producing the stimuli (blowing the bubbles). This result indicates that the validity of some tasks could also depend on the observer’s skills and that particular attention should be given to each step of the procedure, such as obtaining the child’s attention before eliciting a behaviour. Professionals’ fidelity to training in all other steps involved in the four items was high, suggesting that the short training provided is sufficient to transmit the information necessary to use these four items as an augmentative procedure in Ecuadorian paediatric settings.

Finally, the perception of this augmentative procedure was unanimously positive in its adaptation to their daily practice in terms of time, equipment and comfort and on the probability of using it as part of the surveillance of children’s development. However, it should be noted that the responses are not free from a desirability bias as they could have been affected by the researcher’s presence.

The complete assessment of a child suspected to develop ASD may require highly qualified professionals, which can significantly impact waiting times (Zwaigenbaum & Penner, 2018) and result in a high cost for families when health systems have not provided coverage (Durkin et al., 2015). For this reason, access to professional education about the different phases of diagnosis, including anamnesis, interviews with parents and medical examinations of individuals with ASD, must be guaranteed in all paediatric and community-health academic contexts. A brief clinical observation is not designed to provide enough reliable information to formally identify cases of ASD (Gabrielsen et al., 2015). The items tested in this study can be part of an augmentative procedure, aiming to transfer research expertise into clinical practice. The use of an observational procedure specifically designed to monitor socio-communicative behaviours in young children may prove to be well adapted in specific socio-economic contexts where ASD assessments provided by specialists are limited. An important contribution of this approach is that in these contexts, procedures built around universally recognised clinical early signs of ASD could motivate professionals to deepen the investigations using a validated screening to collect information from parents. Firstly, this could reduce unnecessary variability in identification practices in paediatric environments. Secondly, using a short observational procedure at any opportunity professionals come into contact with a child may also prove helpful in identifying false negatives, knowing that some children who do not present difficulties at the beginning of the first year can present them later in development. The implementation of specific training modules for professionals, that follow current recommendations for good practices in medium- and low-income contexts (Buffle & Naranjo, 2021; Divan et al., 2021; Durkin et al., 2015; Nickel & Huang-Storms, 2017), together with the examination of screening procedures and questionnaires, tested by local professionals in a group of children from the general population, could contribute to the provision of quality early identification services that are adapted to the needs of the Ecuadorian population (e.g. Albores-Gallo et al. (2012); Gatica-Bahamonde et al. (2021); Roman- Urrestarazu et al. (2021)).

## Limitations and perspectives

Although our sample represented children from urban and rural regions, we could not recruit participants from distant areas such as the Andean higher plateau or the Amazonian areas that cover 48% of the national territory and where approximately 5% of the population lives. Therefore, our findings could not be generalised to those settings. The perspectives opened by this study are related to increasing the knowledge and sense of self-efficiency among professionals and supporting the generalisation of ASD detection practices in public and private paediatric services. In this context, a further reasonable step would be to test these four tasks in populations with an increased likelihood of developing ASD (siblings) or populations with a diagnosis. It should also be considered that screening and augmentative procedures may not necessarily lead to a referral to a complete assessment or an early intervention programme. Many other factors, such as families not understanding the reason for specialised-follow-up services not being available, justify setting up a regular and generalised early detection programme. Continuing to investigate ways to support professionals in a paediatric setting that support early identification in each context would be essential. Finally, this work opens a perspective to the study of important psychometric indicators, such as specificity and sensitivity, that need to be assessed on a sample of children who end up receiving a diagnosis of ASD.
